# Supplementary material for: Single-cell transcriptome atlas of the human corpus cavernosum
Source: Nat Commun. 2022 Jul 25;13:4302. doi: 10.1038/s41467-022-31950-9 (PMC9314400; doi:10.1038/s41467-022-31950-9)
Supplement: Supplementary file 1 — Supplementary Information [file 41467_2022_31950_MOESM1_ESM.pdf]

Single-cell Transcriptome Atlas of the Human Corpus Cavernosum

LiangYu Zhao et al.

Supplementary Information

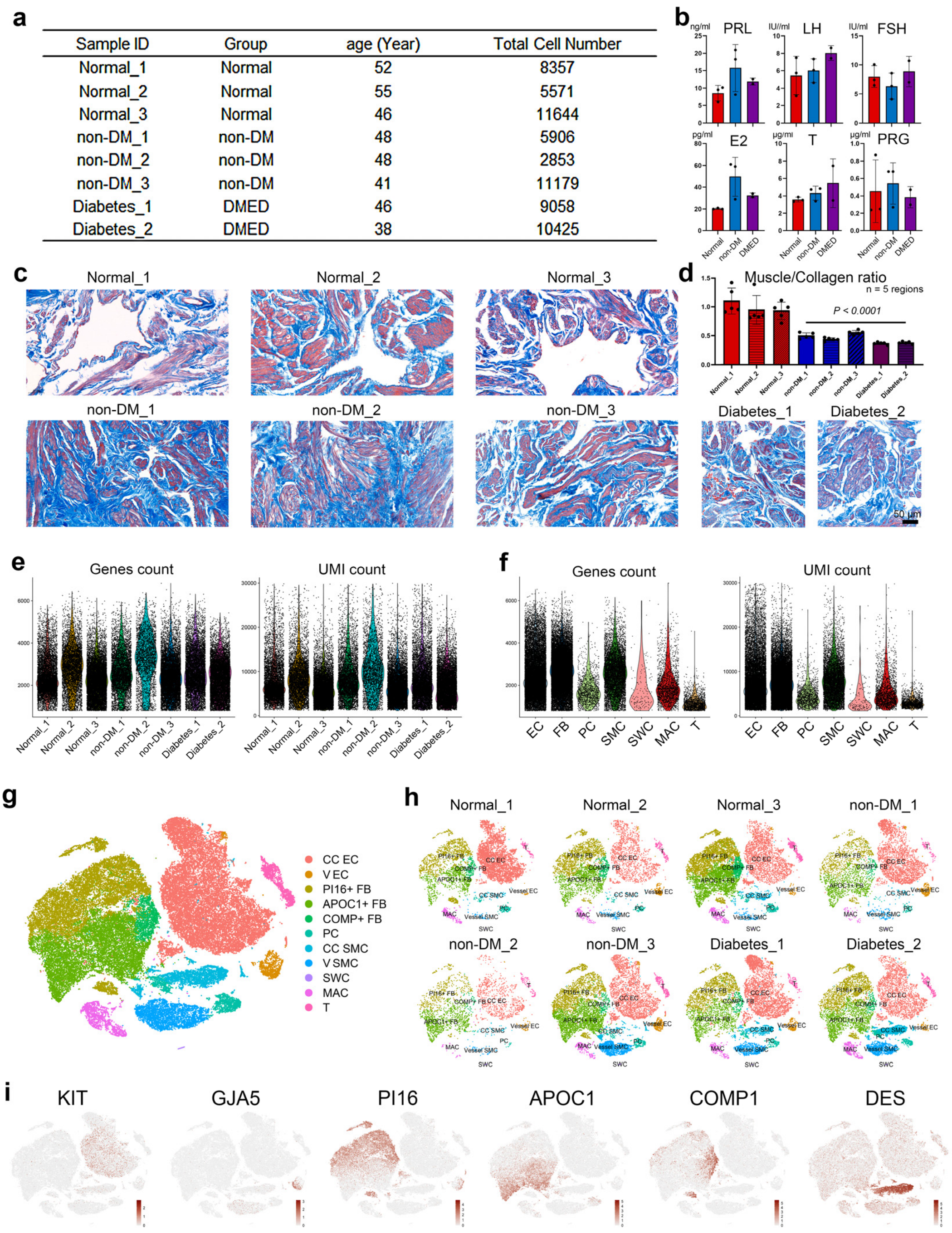

Sample ID	Group	age (Year)	Total Cell Number
Normal_1	Normal	52	8357
Normal_2	Normal	55	5571
Normal_3	Normal	46	11644
non-DM_1	non-DM	48	5906
non-DM_2	non-DM	48	2853
non-DM_3	non-DM	41	11179
Diabetes_1	DMED	46	9058
Diabetes_2	DMED	38	10425

Supplementary Figure 1. Clinical information of enrolled samples and sequencing quality control.

(a) Clinical information of enrolled samples in this study.

(b) Sex hormone levels of normal and ED patients. n = 3 biologically independent samples. T, testosterone; FSH, follicle-stimulating hormone; LH, luteinising hormone; E2, oestradiol. Data are presented as mean values ± SD.

(c-d) The Masson staining of each CC sample. The statistics indicate the ratio of intensity between muscle (red region) and collagen (blue region). Data are presented as mean values ± SD, n = 5 regions. The statistical analysis was made by ANOVA test; two-tailed; the confidence interval is 95%.

(e-f) Violin plot showing the total expressed gene count or read count in each sample or cluster.

(g-h) tSNE plot showing a more detail classification approach where cavernosal trabecular and nearby vessels were distinguished.

(i) tSNE plot showing the expression of marker genes. A gradient of grey to red indicates low to high expression levels.

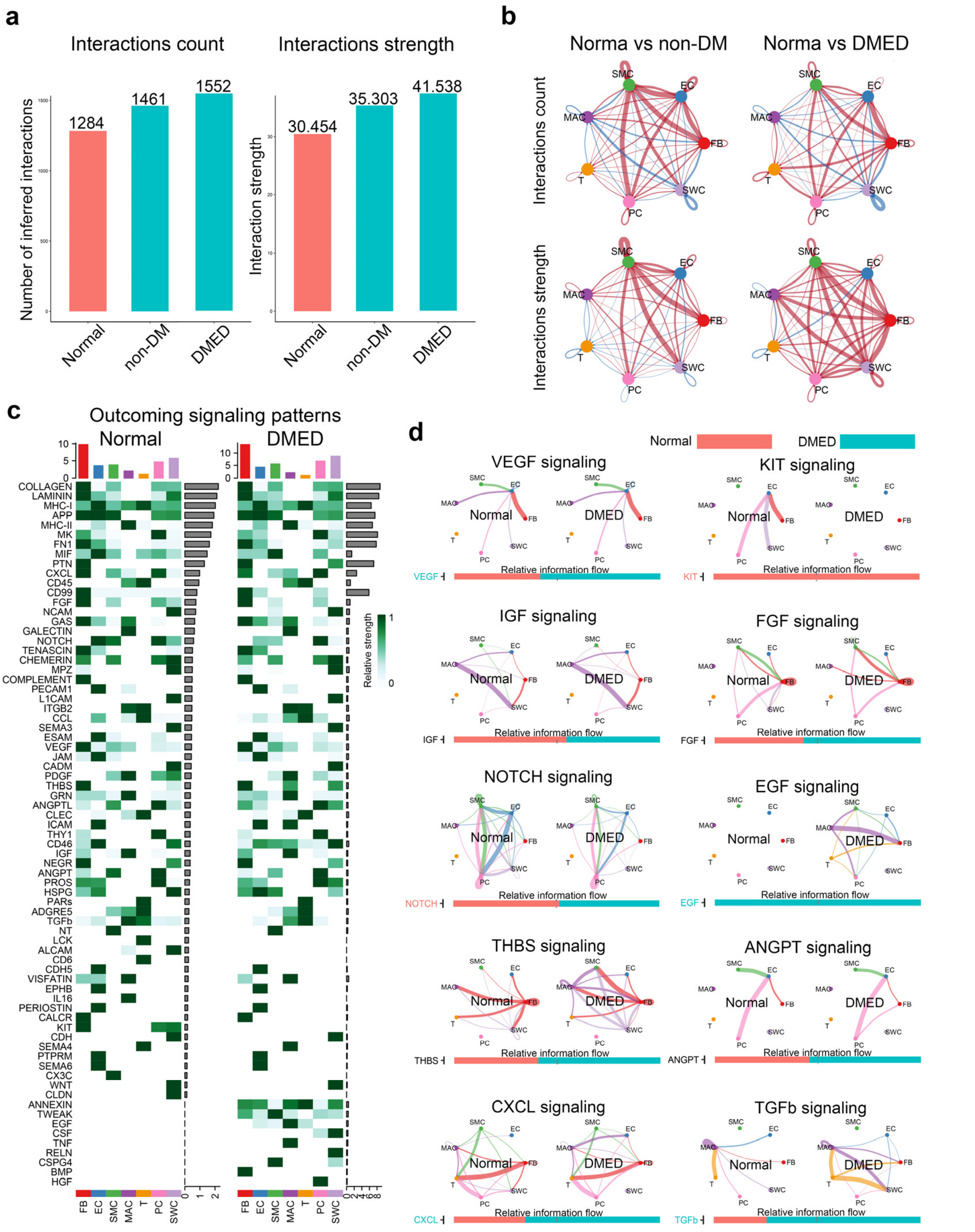

Supplementary Figure 2. Cell–cell communication network within the CC microenvironment.

(a) The barplot showing the total interaction count and strength within CC microenvironment of normal and ED patients.

(b) Circle plots showing the up- or down-regulation of cell–cell communication network between normal and ED patients. The red line represents up-regulated interaction and the blue line represents down-regulated.

(c) Heatmap of the outcoming signals of each cluster between normal and DMED patients. A gradient of white to dark green indicates low to high expression weight values in the heatmap.

(d) Circle plots depict the differential strength (right) of specific signals in the cell-cell communication network between normal and DMED CC. Red or blue edges represent increased or decreased signals in normal state.

a RNA-seq result of FB *in vitro*

gene name	NC_1_count	NC_2_count	NC_1_FPKM	NC_2_FPKM
FGF2	16889	17073	100.20399	100.1620742
FGF7	3382	6337	26.6205705	49.32190155
KITLG	4937	5518	32.5151464	35.93491984
IGF1	6	11	0.04068728	0.073755412
VEGFA	2215	2089	5.60964128	5.231330356
VEGFB	1163	1180	27.0344918	27.12269279
VEGFC	2094	2182	49.0035472	50.49148227
HBEGF	525	491	7.91355639	7.318241785
CXCL12	350	395	3.08641791	3.045539175
TGFB1	2887	2722	42.9891412	40.07859768
TGFB2	184	210	1.69615798	1.692574596
JAG1	3.48552742	3.77330045	675	739
THBS1	134656	135394	726.678698	722.4844644
THBS2	13993	14803	34.2534145	35.83068721
EDN1	37	51	1.13819155	1.551294471

b RNA-seq result of EC *in vitro*

gene name	NC_1_count	NC_2_count	NC_1_FPKM	NC_2_FPKM
FGFR1	726	910	1.06250693	1.164438934
KIT	128	197	1.33378764	1.794833541
IGF1R	1139	1584	5.0004179	6.080195811
IGF2R	4475	5744	9.60787652	10.78273131
FLT1	6443	7416	26.2060873	26.37325448
KDR	9277	11702	80.229276	88.48412224
EGFR	458	544	2.02564366	2.103664902
CXCR4	1327	1418	19.5581068	18.27309054
TGFBR1	77	350	0.31729763	1.261030532
TGFBR2	19209	23762	111.985367	121.1209966
TGFBR3	224	248	1.13433938	1.098059368
NOTCH1	3305	4802	20.0680059	25.49381836
NOTCH2	1339	1899	4.12992797	5.121141208
NOTCH3	18.4270798	18.6709751	3117	3194
EDN1	9393	9953	343.411747	318.1594241

c

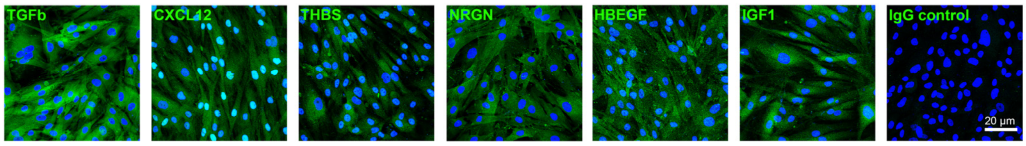

d

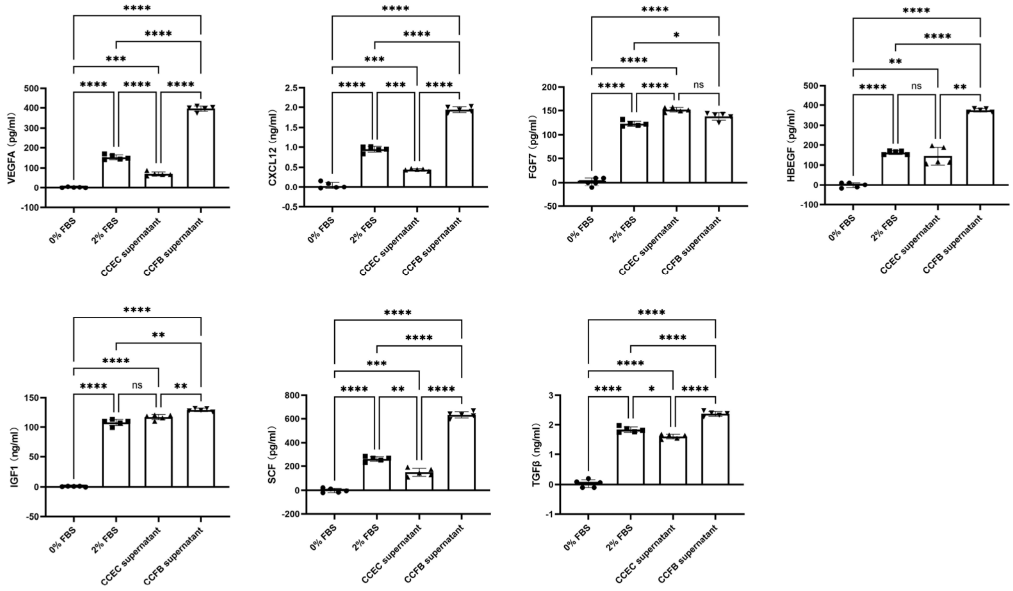

Supplementary Figure 3. The expression pattern of signaling ligands predicted by CellChat in FB.

(a-b) RNA-seq of FB and EC that were cultured *in vitro* showing the expression levels of relevant signal ligands and receptors, respectively.

(c) ICC staining of signal ligands predicated by CellChat in FB *in vitro*. The scale bar represents 20 μm.

(d) ELISA showing the concentration change of signal ligands in EC and FB supernatant. Data were shown as mean ± SD, n = 5 independent experiments. The statistical analysis was made by ANOVA with Tukey's multiple comparisons test; two-tailed; the confidence interval is 95%. *P < 0.05, **P < 0.01, ***P < 0.001.

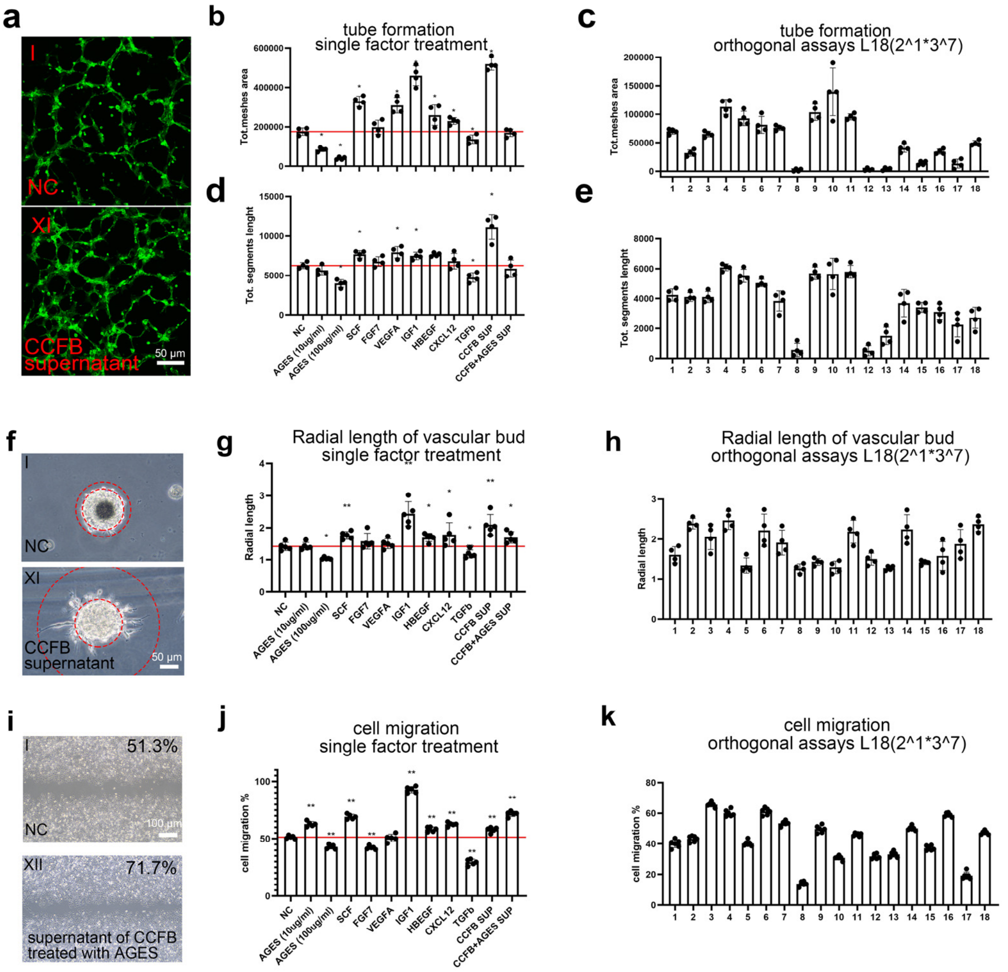

Supplementary Figure 4. CC tissue repair related functional verification after single factor stimulation or the comprehensive effect of multiple factors.

(a-e) Phalloidine staining showing typical changes of EC tube formation in NC and FB supernatant treated group (a). Total segment length and total meshes area score are used to represent the level of tube formation in each group. n = 4 independent experiments.

(f-h) The radial length of EC mass bud change after single factor stimulation or the comprehensive effect of multiple factors. n = 5 (g) or 4 (h) independent experiments.

(i-k) EC migration change after single factor stimulation or the comprehensive effect of multiple factors. n = 4 independent experiments.

All data were shown as mean ± SD. The orthogonal experimental design was listed in Supplementary Table 6. The statistical analysis in (b), (d), (g) and (j) were made by ANOVA with Tukey's multiple comparisons test; two-tailed; the confidence interval is 95%. *$P < 0.05$, **$P < 0.01$, ***$P < 0.001$.

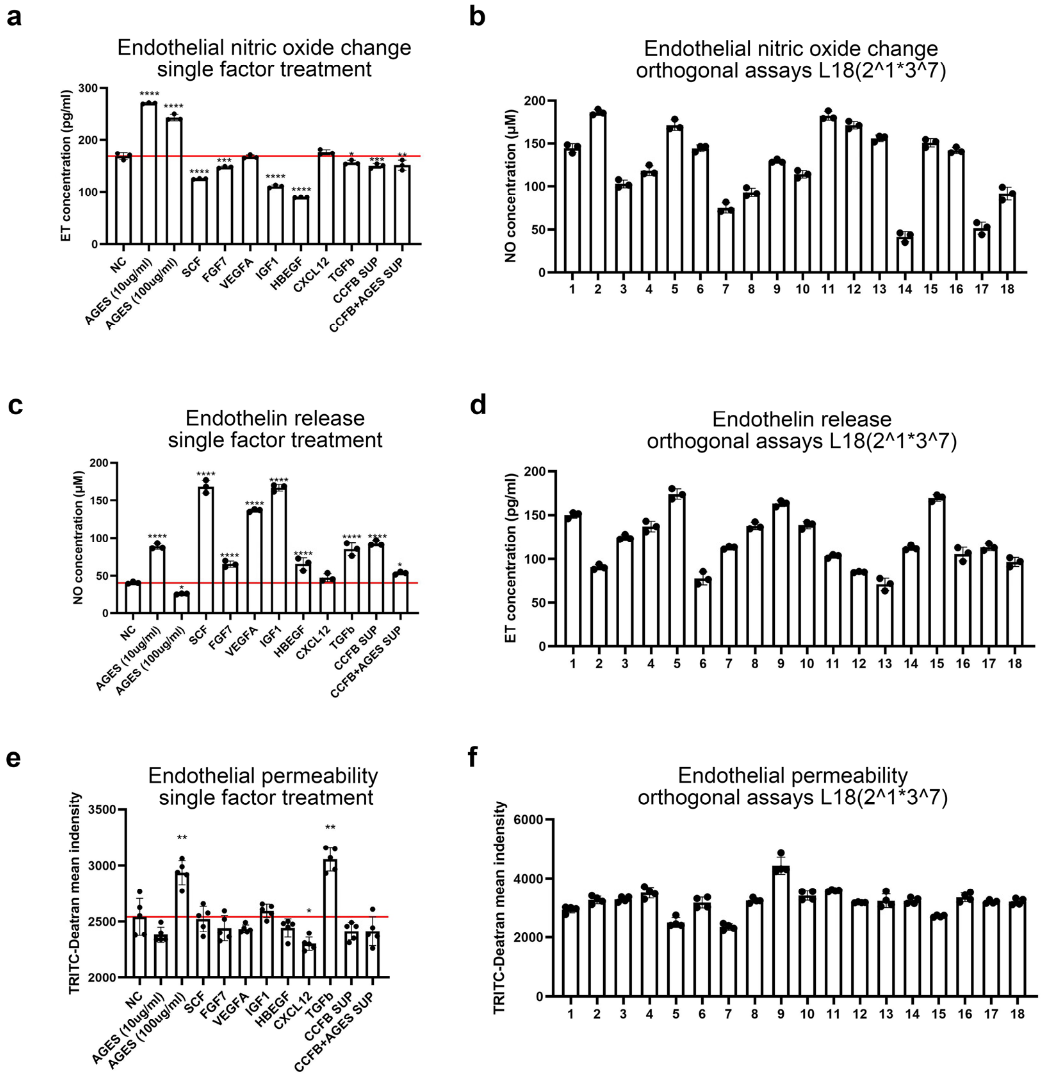

Supplementary Figure 5. Penile erection related functional verification after single factor stimulation or the comprehensive effect of multiple factors.

(a-b) ELISA results showed that the concentration of NO in the supernatant of EC cells cultured under various of stimulation conditions for 24 hours. n = 3 independent experiments.

(c-d) ELISA results showed that the concentration of ET in the supernatant

of EC cells was cultured under various stimulation conditions for 24 hours. n = 3 independent experiments.

(e-f) Fluorescence intensity in Transwell lower pore indirectly indicated the changes of endothelial permeability under various stimulation conditions for 24 hours. n = 5 (e) or 4 (f) independent experiments.

All data were shown as mean ± SD. The orthogonal experimental design was listed in Supplementary Table 6. The statistical analysis in (a), (c) and (e) was made by ANOVA with Tukey's multiple comparisons test; two-tailed; the confidence interval is 95%. *$P < 0.05$, **$P < 0.01$, ***$P < 0.001$.

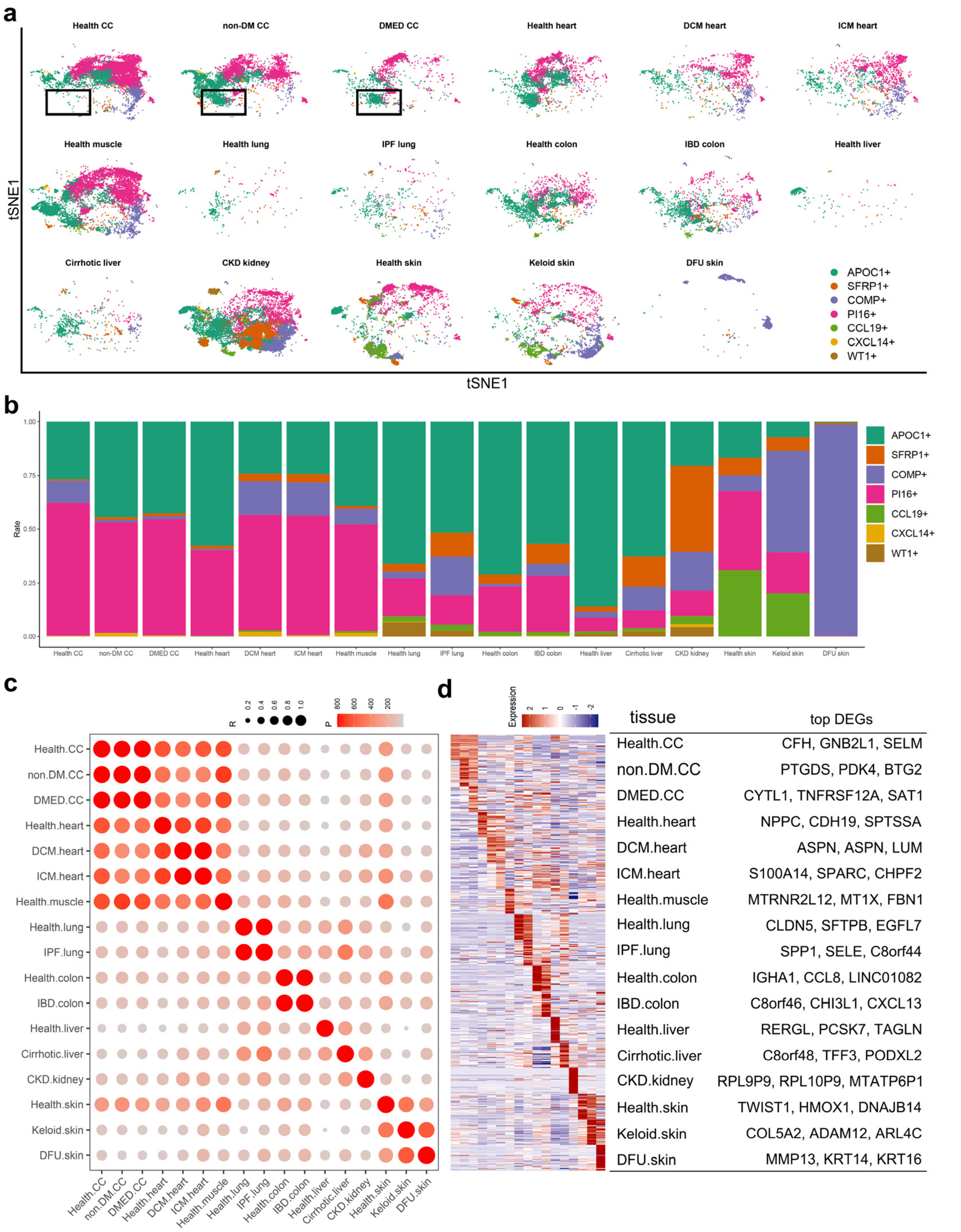

Supplementary Figure 6. Cross-tissue organization of the fibroblast.

(a) tSNE plots of all fibroblast from eight different tissues under normal and disease state. Cells are coloured according to their types.

(b) Bar plot showing the cell count proportion of each FB subcluster in different sample types.

(c) Bubble diagram showing the similarity (correlation) of FB among different sample types. A gradient of light blue to red indicates the P-value. The size of the bubble indicates the correlation coefficient.

(d) Heatmap of the top 30 DEGs in each FB from different sample types. A gradient of light blue to dark red indicates low to high expression levels in the heatmap.

DCM: dilated cardiomyopathy; ICM: ischemic cardiomyopathy; IPF: idiopathic pulmonary fibrosis; IBD: inflammatory bowel disease; CKD: chronic kidney disease; DFU: diabetic foot ulcer.

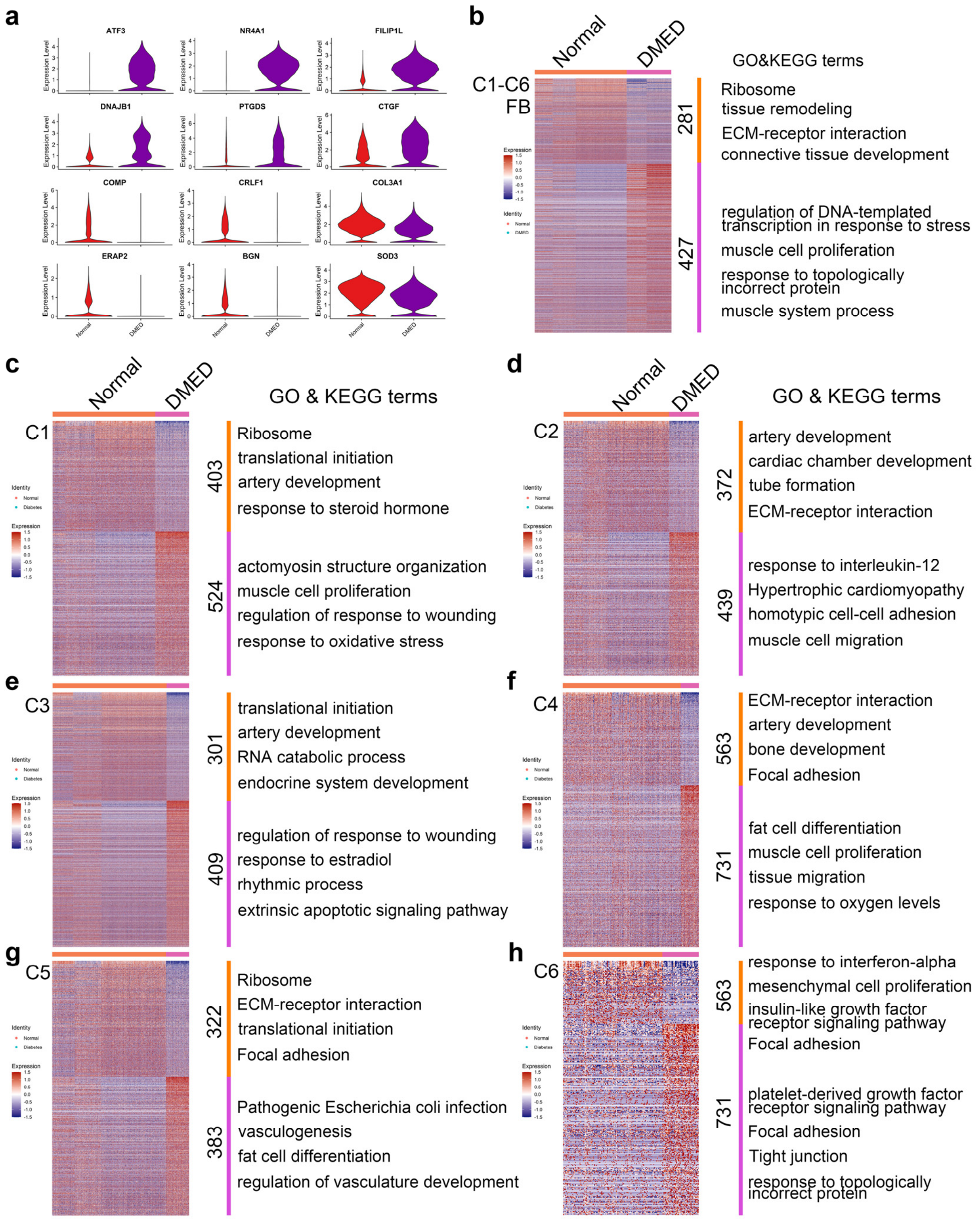

Supplementary Figure 7. the DEGs and functional annotations between normal and DMED FB.

(a) Violin plot showing the different expression of top DEGs between normal and DMED FB.

(b-h) Heatmap showing the DEGs between normal and DMED in all FB

or each FB subclusters, the right panel showed the GO or KEGG enrichment terms.

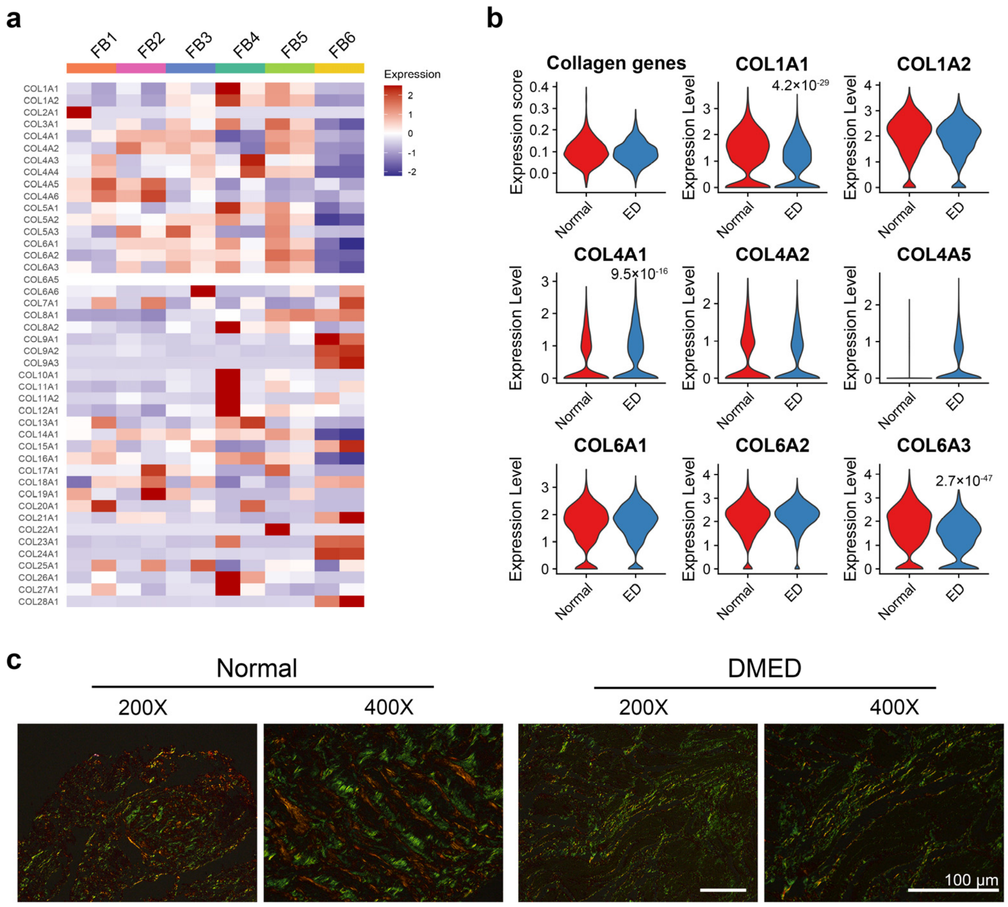

Supplementary Figure 8. The expression pattern of collagen in the six FB subclusters.

(a) Heatmap of the collagen gene expression levels in six FB subclusters. For each subcluster, the left panel represents normal male and the right panel represents DMED FBs. A gradient of dark blue to dark red indicates low to high expression weight values in the heatmap.

(b) Violin plot showing the different expression of collagen I, IV, and VI between normal and DMED FB. *P*-values of the genes with significant

differences are marked. Statistical analysis between one group and the others were made by two-tailed Wilcoxon Rank Sum test in R.

(c) Sirius red staining of normal and DMED CC paraffin sections under polarised light microscopy. Type I collagen fibres are stained strong orange yellow or bright red, type III collagen fibres are stained green, and type IV collagen fibres are stained light yellow.

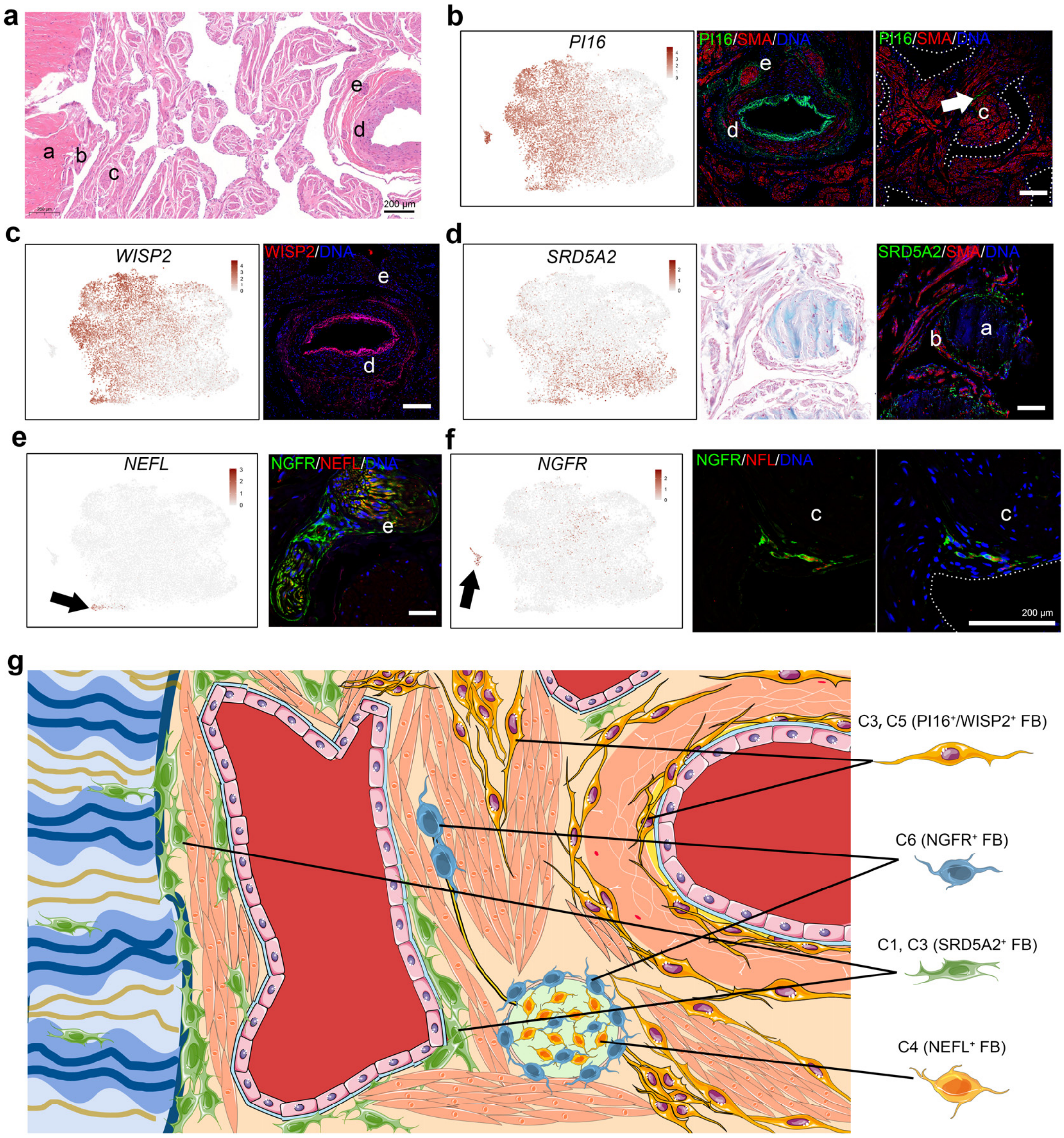

Supplementary Figure 9. The heterogeneity of spatial location in the six FB subclusters.

(a) The major representative regions are labelled in the H&E staining of the CC. a, septum pectiniforme; b, region near septum pectiniforme; c, sinusoid (cavernosal trabeculae); d, cavernosal artery; e, nerve bundles. The scale bar represents 200 μm.

(b–f) The transcription pattern of FB subcluster marker genes (b) PI16, (c) WISP2, (d) SRD5A2, (E) NEFL and (f) NGFR and their protein location in the CC. The middle panel of (d) was Alcian blue staining where the cartilage in septum pectiniforme was blue, and the nuclei were red. The scale bar represents 200 μm.

(g) A sketch map indicating the spatial location of the six FB subclusters in the CC.

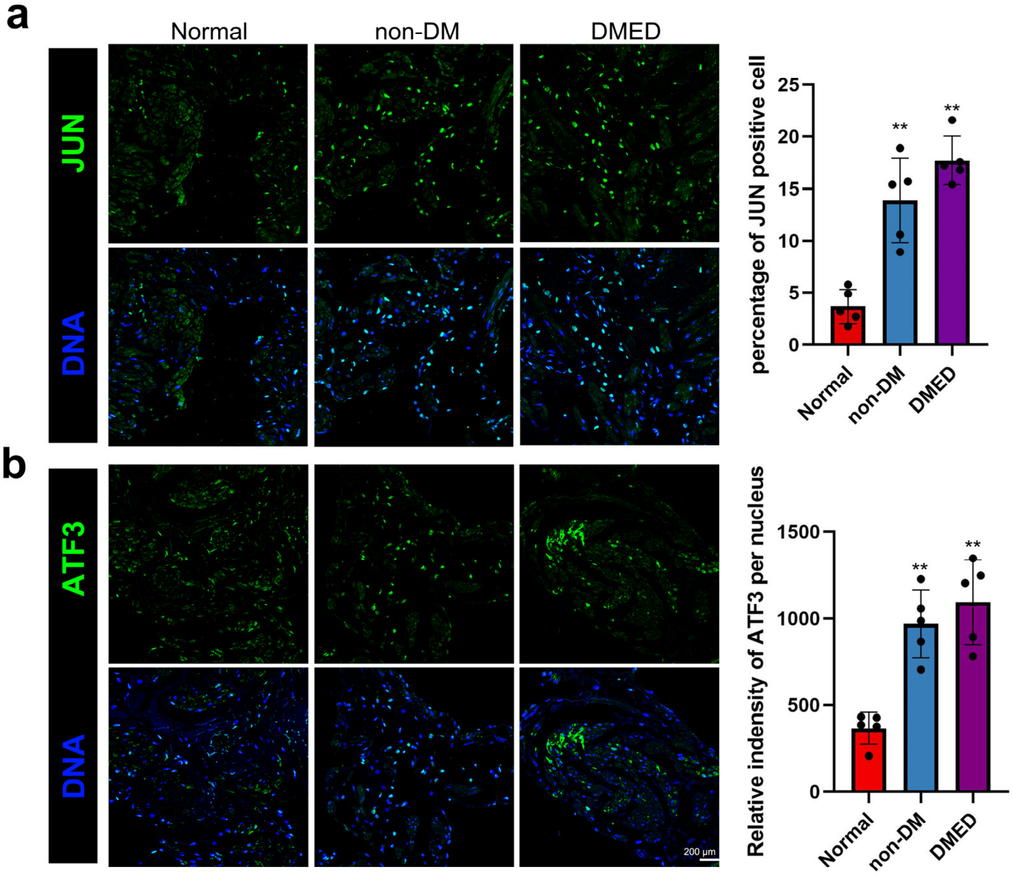

Supplementary Figure 10. The IHC staining of JUN and ATF3 in normal and ED CC tissues.

(a-b) The IHC staining and statistics of JUN and ATF3 in normal and ED CC tissues. The scale bar represents 200 μm. All data were shown as mean ± SD. The statistical analysis was made by Tukey multiple comparisons ANOVA test; n = 5 different regions; two-tailed; the confidence interval is 95%. **P* < 0.05, ***P* < 0.01, ****P* < 0.001.

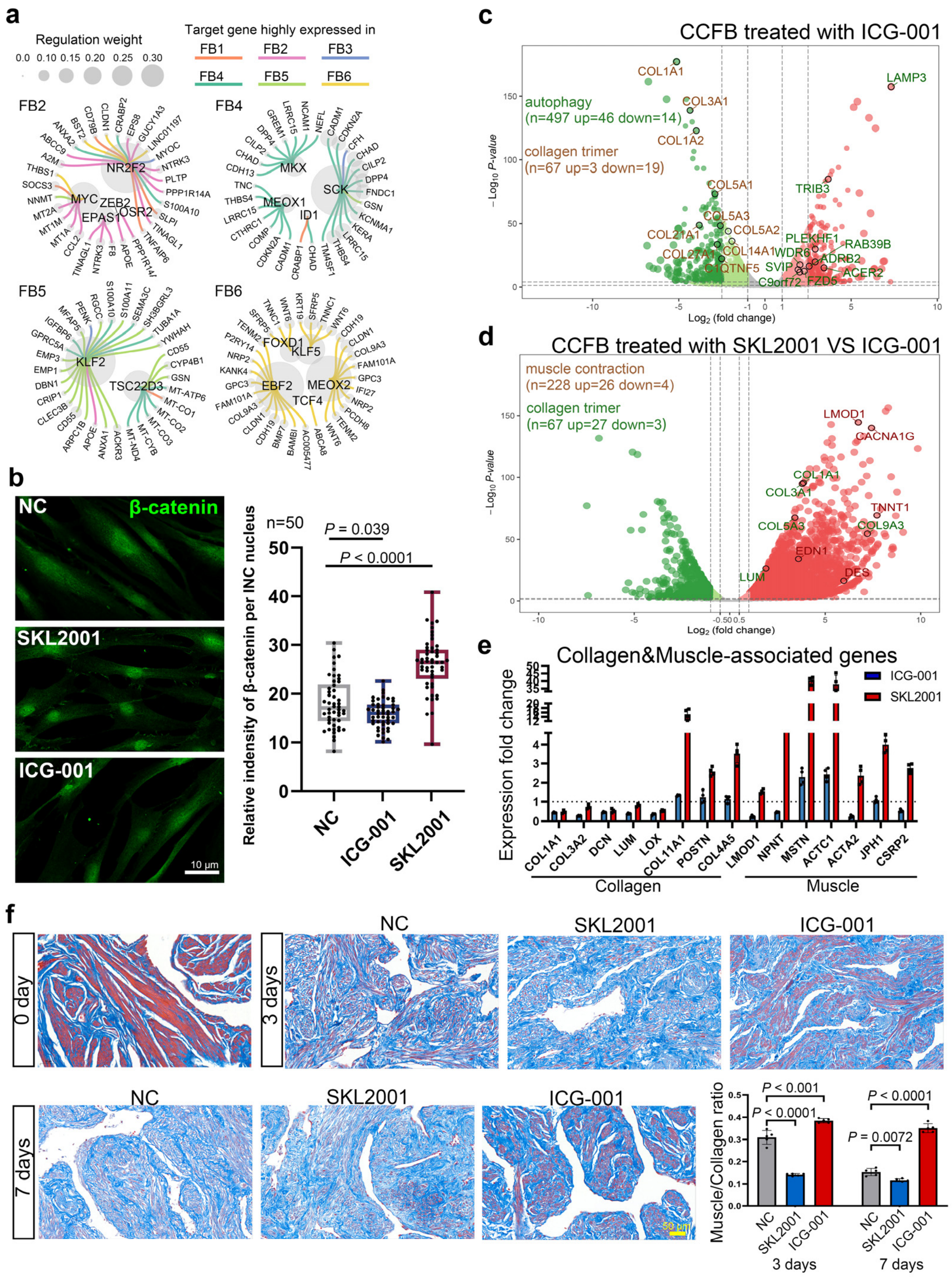

Supplementary Figure 11. The effect of WNT pathway activation on the CCFB *in vitro*.

(a) The top candidate master regulators and their target genes for the FB2, FB4, FB5, and FB6 subclusters. The bubble size indicates the weight from

high to low. The colour of the line indicates in which FB subcluster the target gene was highly expressed.

(b) Immunofluorescence staining of β-catenin (green) in FBs treated with SKL2001 ($P < 0.0001$) or ICG-001 ($P = 0.039$). The scale bar represents 10 μm. Box plots indicate median (middle line), 25th, 75th percentile (box) and 5th and 95th percentile (whiskers) as well as outliers (single points). The statistical analysis was made by ANOVA with Tukey's multiple comparisons test; n = 50 FBs in each group; two-tailed; the confidence interval is 95%.

(c) Volcano plot showing the DEGs between FBs treated with ICG-001 and negative controls by RNA sequencing. Genes belonging to the GO terms "autophagy" and "collagen trimer" were labelled with green and brown, respectively.

(d) Volcano plot showing the DEGs between FBs treated with SKL2001 and ICG-001 by RNA sequencing. Genes belonging to the GO terms "muscle contraction" and "collagen trimer" were labelled with violet and green, respectively.

(e) qPCR results showing the expression fold change of collagen- and muscle-associated genes in FBs with SKL2001 or ICG-001 treatment. The gene expression levels of normal FBs without SKL2001 or ICG-001 treatment (DMSO treated) were used as the baseline values. n = 4 technical repeats. Data are presented as mean values ± SD. The statistical analysis

was made by ANOVA with Tukey's multiple comparisons test; two-tailed; the confidence interval is 95%.

(f) The Masson staining of CC tissue treated with SKL2001 or ICG-001 for 0–7 days *in vitro*. The statistics indicate the ratio of the intensity between muscle (red region) and collagen (blue region) under 5 different regions. The scale bar represents 50 μm. Data are presented as mean values ± SD. The statistical analysis was made by ANOVA with Tukey's multiple comparisons test; two-tailed; the confidence interval is 95%.

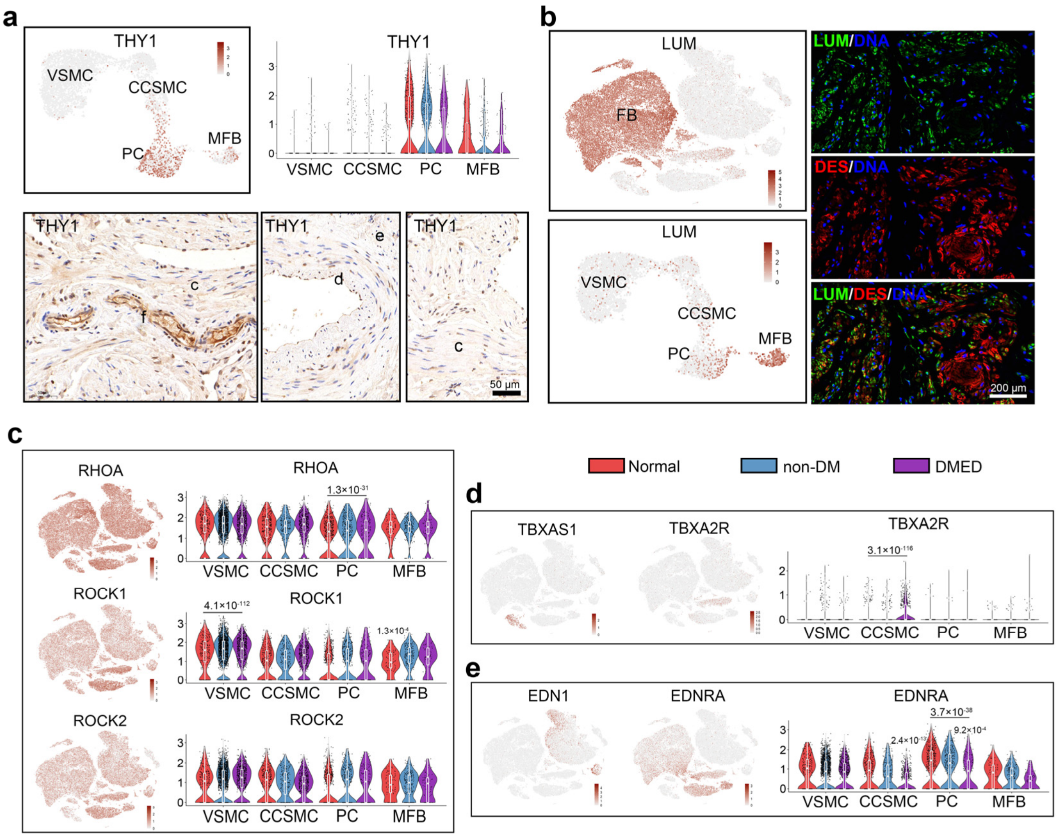

Supplementary Figure 12. The heterogeneity of the SMC subclusters and the transcription difference between normal and ED SMC.

(a) The transcription pattern of *THY1* in the four SMC subclusters (upper panel). Immunohistochemical staining of THY1 in CC paraffin sections (bottom panel) showing strong positive staining around small blood vessels. The scale bar represents 200 μm. Box plots indicate median (middle line), 25th, 75th percentile (box) and 5th and 95th percentile (whiskers) as well as outliers (single points).

(b) The transcription pattern of *LUM* in all cavernosal cells and in the four SMC subclusters (upper panel). Immunohistochemical co-staining of LUM (green) and DES (red) in CC paraffin sections (bottom panel). The arrow marks a region of arteriole. The scale bar represents 200 μm.

(c) Bar plot of the top activated/inhibited IPA signalling pathways (left panel) and cell function (right panel) based on the DEGs between the VSMC and CCSMC subclusters. A gradient of dark blue to dark red indicates inhibition to activation of the term. Statistical analysis was based on Fisher's exact test; two-tailed; the confidence interval is 95%.

(d–f) The heterogenic expression pattern of smooth muscle contraction - correlated gene (d) RHOA, (e) prostaglandin H2 and (f) endothelin pathway in the four SMC subclusters. The statistical analysis was made by Wilcoxon (Mann-Whitney) rank sum test; two-tailed; the confidence

interval is 95%. *P*-values with a horizontal line on the violins represent statistical differences between this subcluster and the other subclusters within the SMC cluster. *P*-values without a line on the violins represent a significant difference between this disease type and the two other types within one subcluster. Box plots indicate median (middle line), 25th, 75th percentile (box) and 5th and 95th percentile (whiskers) as well as outliers (single points).

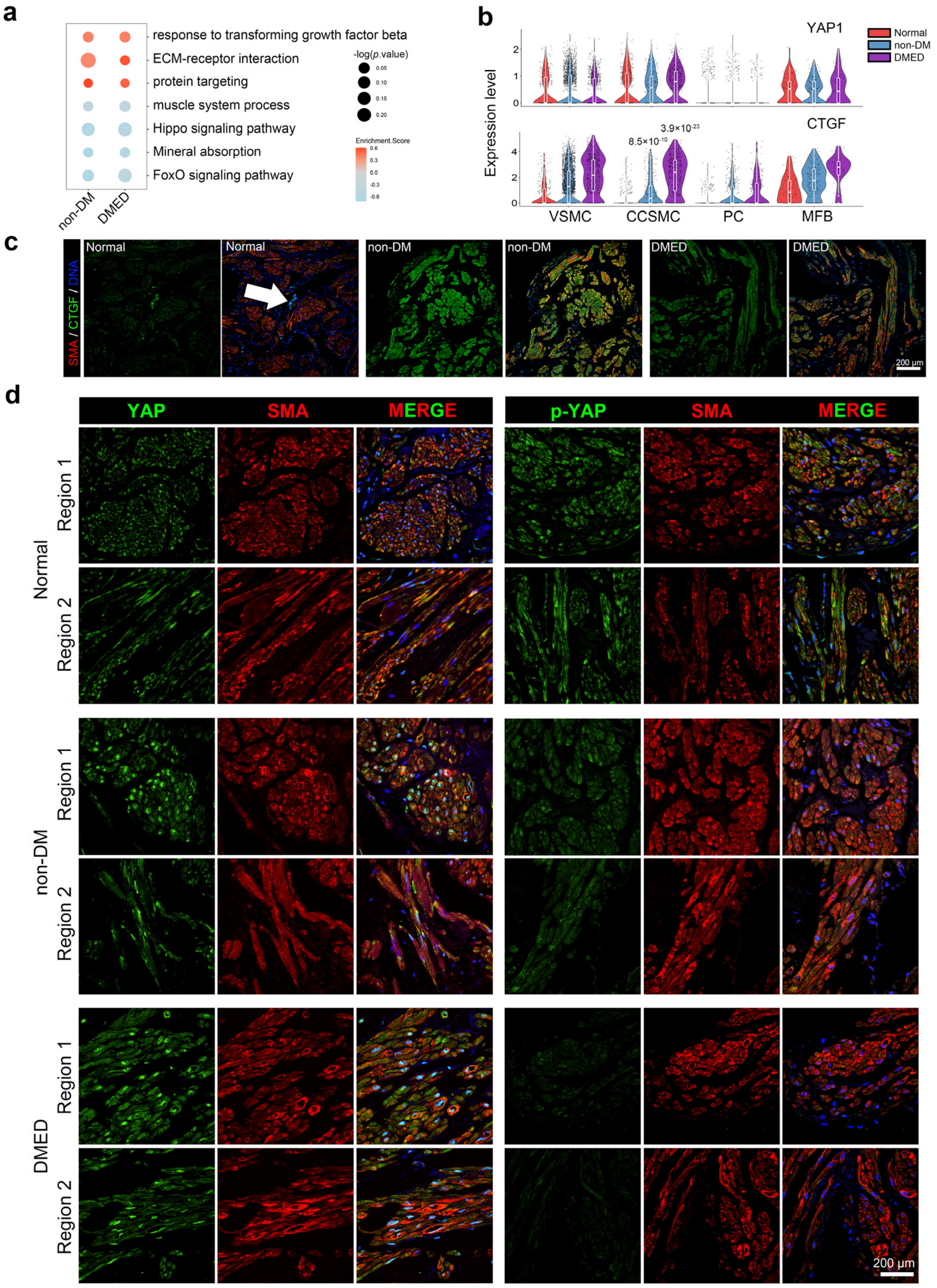

Supplementary Figure 13. The overactivation of YAP and CTGF signal in ED patients SMC.

(a) Bubble diagram of the top activated and inhibited IPA pathways based on the DEGs between non-DM or DMED SMCs compared with normal

SMCs, we can find the Hippo signaling was inhibited in both non-DM and DMED CC. Statistical analysis was based on Fisher's exact test; two-tailed; the confidence interval is 95%.

(b) Violin plot combined with box plot showing the transcription levels of YAP1 in each SMC subcluster between normal male and ED patients. Box plots indicate median (middle line), 25th, 75th percentile (box) and 5th and 95th percentile (whiskers) as well as outliers (single points).

(c) IHC co-staining of CTGF (green) and SMA (red) in normal and ED corpora cavernosa paraffin sections (bottom panel). The scale bar represents 200 μm.

(d) IHC co-staining of YAP/p-YAP (green) and SMA (red) in normal and ED corpora cavernosa paraffin sections (bottom panel). Since the nuclei of CCSMC are elongated oval, in order to more clearly observe the expression of YAP in the nuclei, we selected the short-axis (Region 1) and long-axis (Region 2) cross-sections of the nuclei for observation. The scale bar represents 200 μm.

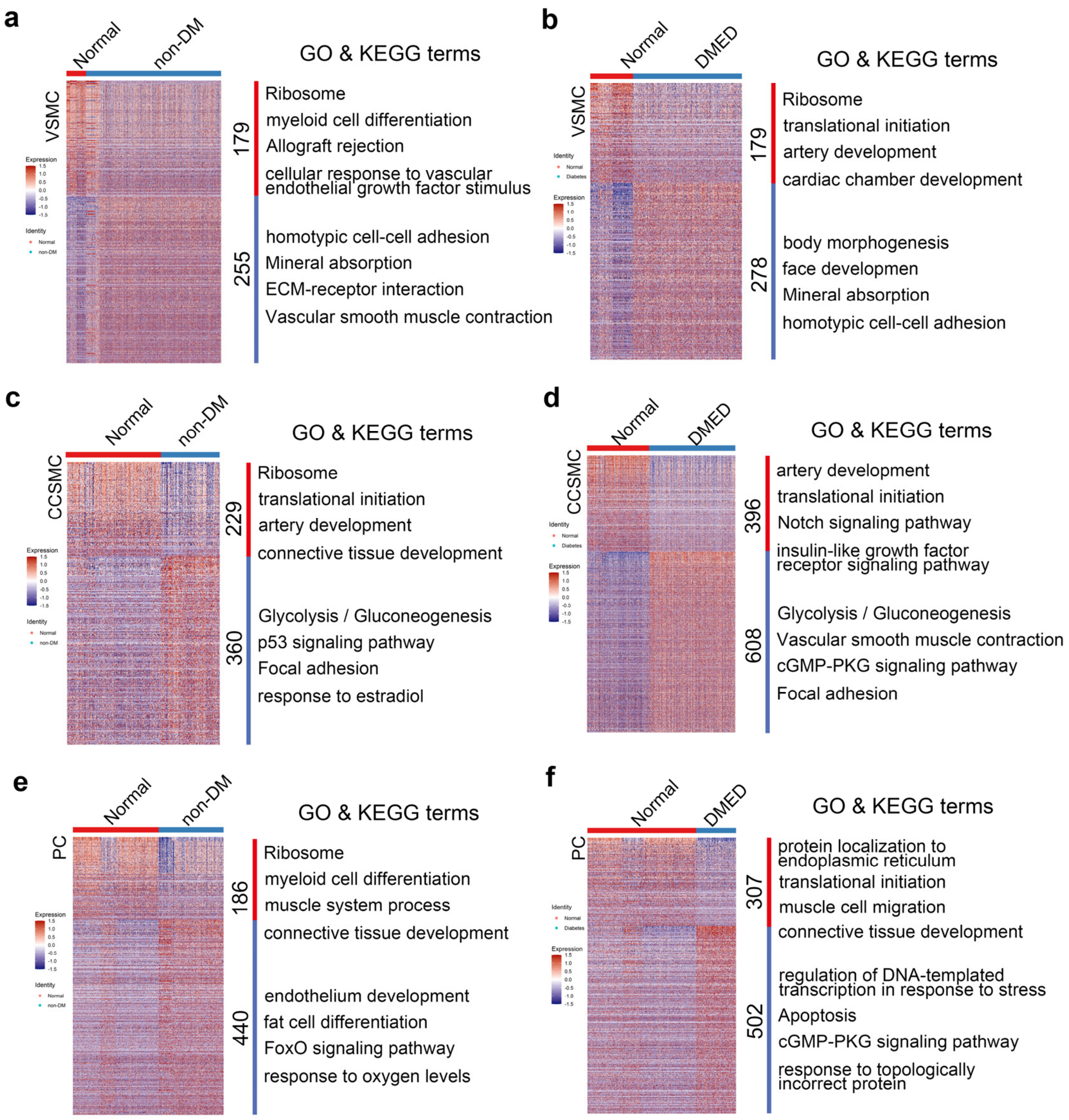

Supplementary Figure 14. the DEGs and functional annotations between normal and ED SMC.

(a-f) Heatmap showing the DEGs between normal and non-DM (a, c, e) or DMED (b, d, f) in each SMC subclusters, the right panel showed the GO or KEGG enrichment terms.

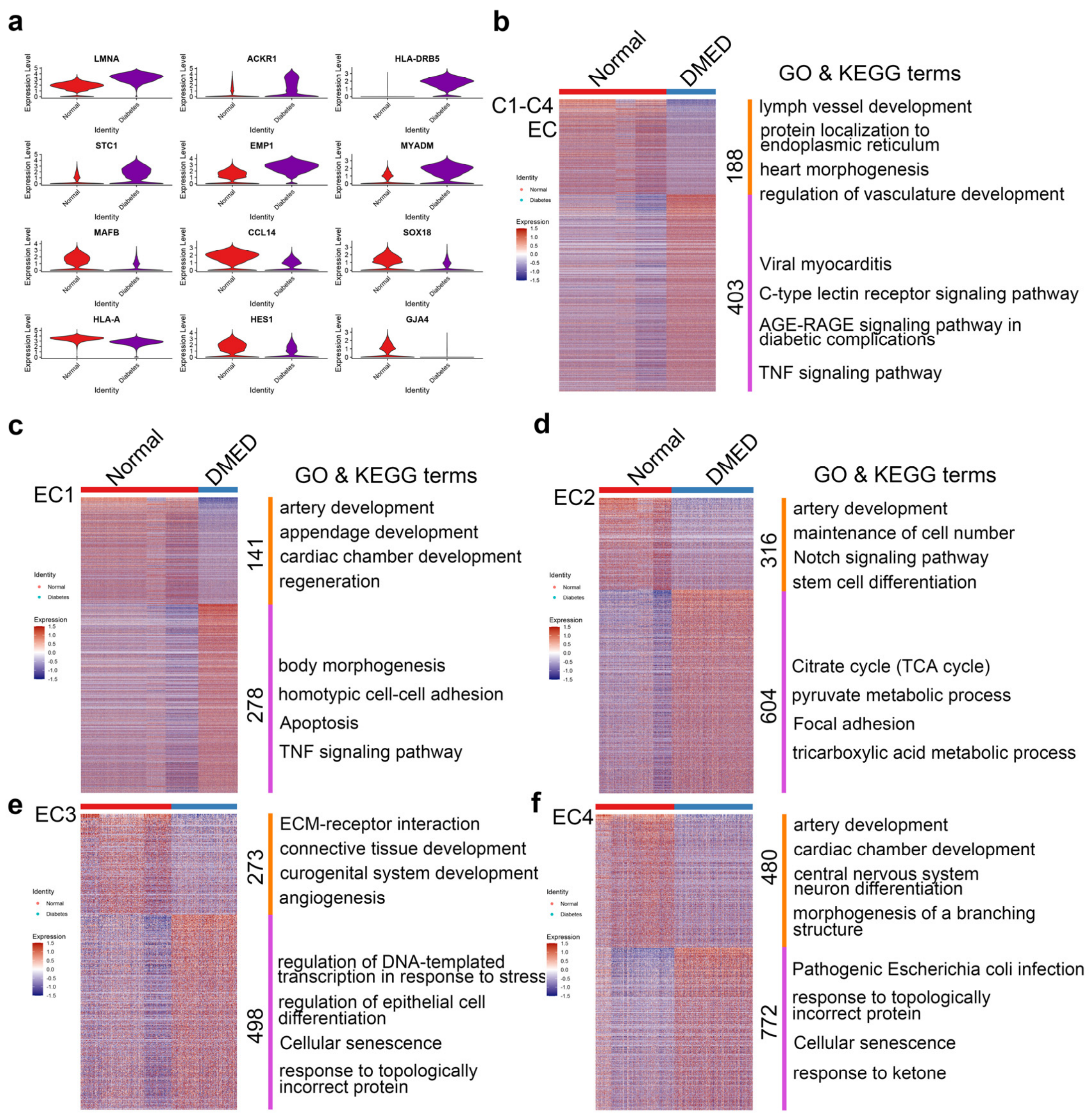

Supplementary Figure 15. the DEGs and functional annotations between normal and DMED EC.

(a) Violin plot showing the different expression of top DEGs between normal and DMED EC.

(b-h) Heatmap showing the DEGs between normal and DMED in all EC or each EC subclusters, the right panel showed the GO or KEGG enrichment terms.

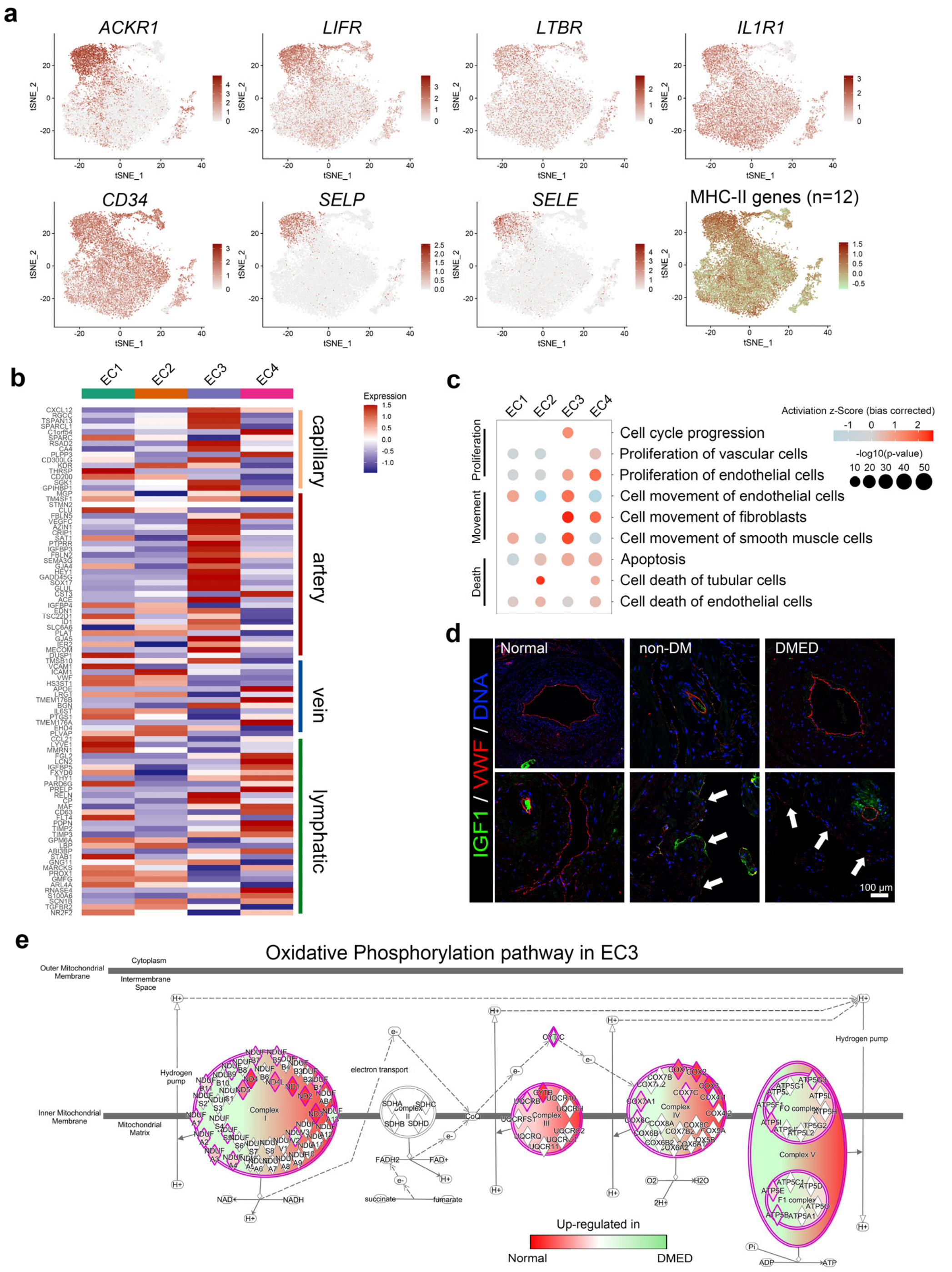

Supplementary Figure 16. The heterogeneity of the EC subcluster.

(a) tSNE plots showing the transcription pattern of immune response genes in the EC cluster.

(b) Heatmap showing the expression levels of the top DEGs that belong to

different endothelial phenotypes in the four EC subclusters. These DEGs were obtained from the Single-Cell Transcriptome Atlas of Murine Endothelial Cells study [27], and genes with specific high expression patterns in at least five tissues were selected. A gradient of dark blue to dark red indicates low to high expression weight values in the heatmap.

(c) Cell function (proliferation and death) predicted by IPA analysis based on the DEGs of each EC subcluster are listed as a bubble diagram. A gradient of light blue to red indicates inhibition to activation of the term. The bubble size indicates the *P*-values from high to low.

(d) Immunohistochemical co-staining of IGF (green) and VWF (red) in normal and ED CC paraffin sections. The upper panels are the region of cavernous artery and its branches, and the bottom panels are the cavernosal trabecular region. The arrow marks the cavernosal trabecular region with endothelial injury. The scale bar represents 100 μm.

(e) Diagram of the oxidative phosphorylation signalling pathway according to the DEGs of the EC3 subcluster between normal and ED patients. A gradient of red to green indicates low to high expression levels of the ED EC3 subcluster in the heatmap.

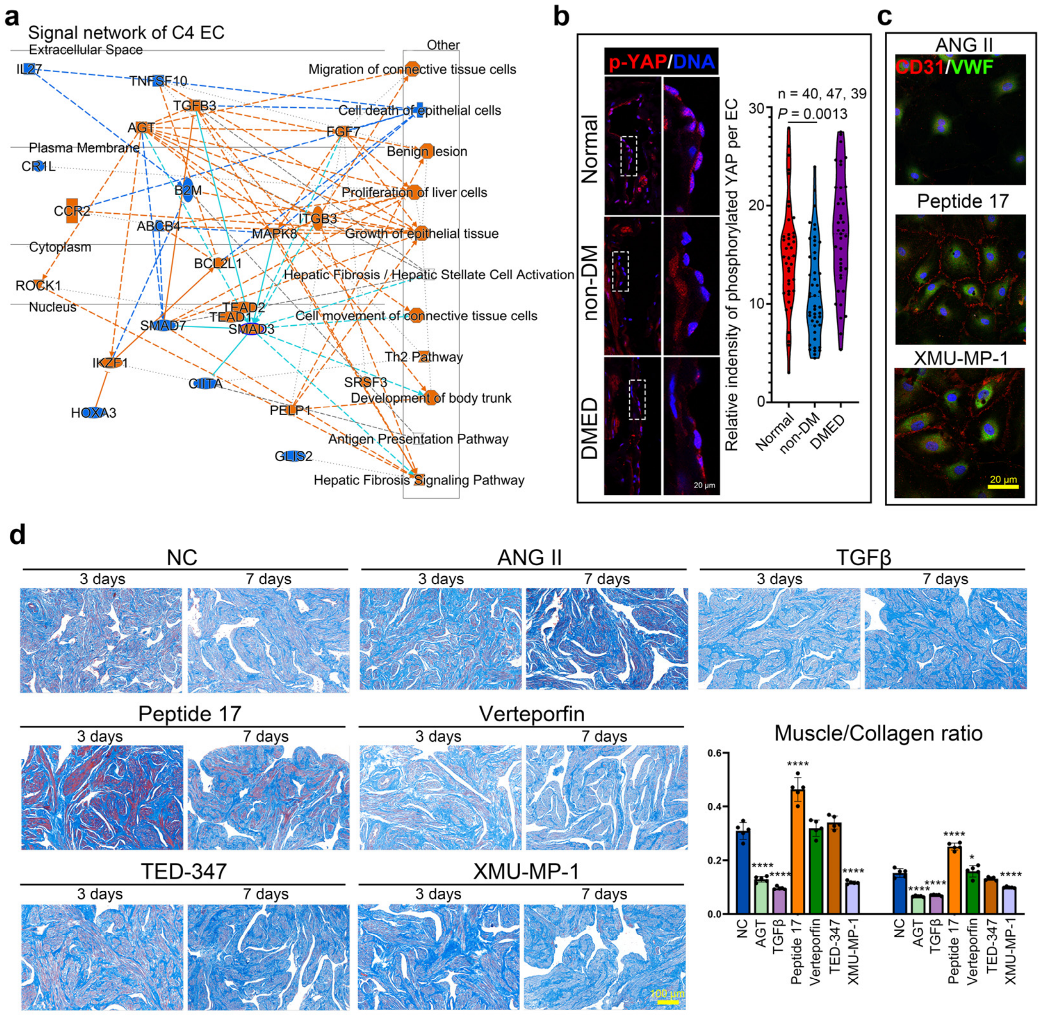

Supplementary Figure 17. Inhibition of YAP alleviated EC and CC tissue injury *in vitro*.

(a) The signalling pathway network of the EC4 subcluster. The regulators are coloured blue or orange based on whether they were predicted to be activated or suppressed, respectively, by IPA analysis.

(b) Immunofluorescence staining of phosphorylated YAP (red) in CC paraffin sections. The middle panel is an enlargement of the dotted box in the left panel. The statistical analysis was made by ANOVA with Tukey's

multiple comparisons test based on 40, 47 and 39 ECs; two-tailed; the confidence interval is 95%.

(c) Immunofluorescence co-staining of CD31 (red) and VWF (green) in ECs treated with ANG II, Peptide 17 (YAP inhibitor), or XMU-MP-1 (MST1/2 inhibitor which could activate YAP protein) *in vitro*. The scale bar represents 20 μm. ANG II, Angiotensin II.

(d) Masson staining of CC tissue treated with ANG II, TGF-β, Peptide 17, Verteporfin, TED-347, or XMU-MP-1 for 0–7 days *in vitro*. The statistics indicate the ratio of intensity between the muscle (red region) and collagen (blue region). The scale bar represents 200 μm. Data are presented as mean values ± SD from three low-power fields. The statistical analysis was made by ANOVA with Tukey's multiple comparisons test; n = 5 different regions; two-tailed; the confidence interval is 95%. $*P < 0.05$, $**P < 0.01$, $***P < 0.001$.
