# Supplementary material for: Single-cell transcriptome atlas of the human corpus cavernosum
Source: Nat Commun. 2022 Jul 25;13:4302. doi: 10.1038/s41467-022-31950-9 (PMC9314400; doi:10.1038/s41467-022-31950-9)
Supplement: Supplementary file 3 — Description of additional Supplementary File [file 41467_2022_31950_MOESM3_ESM.pdf]

Descriptions of Additional Supplementary Data Files

Supplementary Data 1 the clinical information

Supplementary Data 2 the DEGs of 7 major clusters in normal CC

Supplementary Data 3 the DEGs of 11 CC subclusters

Supplementary Data 4 the DEGs between ED and Normal in 7 CC major clusters

Supplementary Data 5 the DEGs between ED and Normal in 11 CC subclusters

Supplementary Data 6 Orthogonal experimental design and statistics results

Supplementary Data 7 the information of FB dataset of other tissues

Supplementary Data 8 the DEGs of 6 CCFB subclusters in health state

Supplementary Data 9 the DEGs between ED and Normal in 6 FB subclusters

Supplementary Data 10 the DEGs of CCFB treated with ICG001 or SKL2001

Supplementary Data 11 the DEGs of 4 SMC subclusters in health state

Supplementary Data 12 the DEGs between ED and Normal in 4 SMC subclusters

Supplementary Data 13 the DEGs of 4 EC subclusters in health state

Supplementary Data 14 the DEGs between ED and Normal in 4 EC subclusters

Supplementary Data 15 RESOURCES TABLE
